# Refining the Make My Day stroke prevention intervention for primary healthcare through co-creation with stakeholders

**DOI:** 10.1186/s40900-025-00676-5

**Published:** 2025-02-06

**Authors:** Cecilia Johnsson, Elin Jakobsson, Maria Hagströmer, Susanne Guidetti, Ann-Helen Patomella, Eric Asaba

**Affiliations:** 1 Department of Neurobiology, Care Sciences and Society, Division of Occupational Therapy, Karolinska Institutet, 141-52 Stockholm, Sweden; 2 Stockholm Gerontology Research Center, 113-46 Stockholm, Sweden; 3 Unit for Research, Development, and Education, Stockholm’s Sjukhem Foundation, 112-19 Stockholm, Sweden; 4 Department of Neurobiology, Care Sciences and Society, Division of Physiotherapy, Karolinska Institutet, 141-83 Stockholm, Sweden; 5 Women’s Health and Allied Health Professionals Theme Medical Unit Occupational Therapy and Physiotherapy, Karolinska University Hospital, 171-76 Stockholm, Sweden; 6 Academic Primary Health Care Centre, Region Stockholm, 104-31 Stockholm, Sweden

**Keywords:** Co-creation, Stroke prevention intervention, Engaging everyday activities, Primary healthcare, Stakeholder engagement, Make My Day

## Abstract

**Objectives:**

To describe and explore the refinement of a stroke prevention intervention and conditions for implementation in primary healthcare by utilising co-creation with stakeholders.

**Method:**

This was an iterative co-creation process of five collaborative workshops engaging stakeholders; healthcare professionals (HP), and persons at risk for stroke, who participated in or delivered a stroke prevention intervention in primary healthcare.

**Results:**

Through co-creation with stakeholders key components for revision were identified in the Make My Day intervention. *The overall pedagogics*, which was recognised as overarching, and three additional key components: *the HP education*, *the intervention sessions*, and *the digital tool* were identified. Moreover, the co-creation process rendered refinements of the prevention program addressing the key components representing stakeholder experiences. Refinements encompass delivering and receiving the Make My Day intervention, material ownership, and the interprofessional team.

**Conclusions:**

The co-creation process revealed the importance of applying a strategic pedagogic approach in a complex intervention. The process underscored the need to augment a sense of material ownership and to improve interprofessional collaboration in primary healthcare, ultimately enhancing the intervention experience and facilitating the change process for individuals at risk of stroke. Utelising a co-creation process in this current intervention allowed for creation of refinements to the intervention optimising conditions for implementation.

## Abstract (plain language summary)

The goal of this project was to improve a stroke prevention program in primary healthcare settings. To do this, we worked closely with stakeholders, ie. healthcare professionals and people at risk for stroke. Through co-creative workshops, we identified important areas on which to focus, such as education strategies, intervention session design, and utility of digital tools. By engaging stakeholders in intervention design and refinement co-creation processes, the program can be more responsive to the needs of the people it serves and optimising a current intervention and the conditions for implematation into practice. We found that taking a strategic approach and promoting teamwork among healthcare professionals can greatly enhance the experience and outcomes of the stroke prevention program.

## Article summary: Strengths and limitations of this study

- The co-creation process utilised the voices of a mix of stakeholders.
- An iterative analysis with stakeholders generated results representative of the stakeholders. -Co-creation in the later stages of the research process optimised conditions for implementation -However, no evaluation of the co-creation process was performed.
- Co-creation in the later stages of the research process optimised conditions for implementation.
- However, no evaluation of the co-creation process was performed.

## Introduction & background

Several methods and guidelines are available to develop, evaluate, and optimise relevance and acceptability related to different healthcare interventions. In order to successfully work with healthcare interventions targeting health, lifestyle, and behaviour change, engagement from stakeholders through all stages of the research process are recommended [15, 33, 42]. Stakeholders include those who deliver and receive the intervention and gatekeepers such as managers and decision-makers who contribute to the research process by sharing their experiences and knowledge [46]. In recent years, more focus has been shed on ways in which to involve stakeholders in a co-creation process [40, 46]. Engaging stakeholders to tailor interventions is needed to ensure that individual needs are met within their specific context [24]. In this paper, the authors will draw on the UK Medical Research Council (MRC) guidelines [46] and, in particular, focus on co-creation in refining a stroke prevention intervention called Make My Day (MMD) [34]. Insights and lessons from engaging stakeholders in refining the MMD intervention can inform development in future interventions. It is important to identify and address potential barriers through the continuum of the research process stages in order to ensure that the intervention is aligned with implementation [15]. Failing to remain open to change may result in reduced effectiveness or even failure to implement an intervention [33]. Engaging stakeholders in the research process is thought to increase the sustainability and relevance of evidence-based interventions [43]. Moreover, collaboration between researchers and stakeholders can bridge the research- and theory-to-practice gap [13], translate theory into the development of an intervention [15], and optimise relevance, acceptability for implementation [42]. Therefore, engaging stakeholders in refining the MMD intervention prior to implementation is crucial to opimise future implemetation.

### Stroke risk and lifestyle

Stroke incidence is projected to increase worldwide regardless of gender and in all age groups between 2020 and 2030 [41]. Even though the prevalence of stroke has decreased in the Western world [5] and in Sweden in the past decades, it is still the third leading cause of death in Sweden [47], and with a high prevalence of unhealthy lifestyle habits [11]. Stroke can have a detrimental impact economically and on everyday life for those involved [52]. Risk factors for stroke include, age, race, socioeconomic factors, and diseases such as high blood pressure, diabetes, and heart disease [5]. However, the cause of 80% of strokes can be attributed to modifiable risk factors, that is, risk factors that are directly related to peoples lifestyle habits and that can be changed [21, 53]. Lifestyle factors such as insufficient physical activity, unhealthy eating habits, hazardous alcohol consumption, and tobacco use contribute to risk of cardiovascular disease and stroke [5, 10]. Addressing these lifestyle habits and risk factors can significantly decrease the risk of stroke [20], with far-reaching impacts on individuals, society, and the economy (World Health Organization, 2021). Yet, current clinical practice guidelines lack structured prevention interventions that effectively address lifestyle habits [2, 37]. To enhance awareness of the impact lifestyle habits have on health and disease and motivate lifestyle change, lifestyle interventions need to be relevant and acceptable to those receiving and delivering the intervention [3, 20]. Socially situated health challenges may be effectively tailored to specific interventions through co-creation with stakeholders [25, 40]. Co-creation has been utilised primarily in the earlier development phases of interventions targeting lifestyle-related health issues [4, 6, 19, 25, 30, 49, 50]. However, engaging stakeholders also in the later stages of the research process was thought imperative to optimise implementation of lifestyle-related interventions [6, 49].

### The Make My Day prevention intervention

Make My Day (MMD)—the intervention evaluated in this study—is a multi-factorial lifestyle-oriented stroke prevention intervention. MMD was initiated in 2014 following the original MRC guidelines [8] and now the updated MRC guidelines [46]. The structure of MMD aims to facilitate and promote a supportive environment for persons at risk for stroke in their change process towards healthier lifestyle habits, reducing stroke risk. In MMD, engaging in everyday activities is regarded as key in the change process influencing one's lifestyle choices and health. MMD has been developed in primary healthcare in the Stockholm Region, Sweden, led by an interprofessional rehab team (occupational therapist, physiotherapist, and dietician) supporting persons at risk for stroke in a lifestyle change process [34]. Prior to delivering the MMD intervention, the healthcare professionals (HP) attended an interprofessional MMD HP education. The education, described in detail elsewhere [17], had a collaborative learning model and peer coaching pedagogy [22, 44] with access to an e-learning platform (The Canvas website). The MMD prevention intervention is delivered to a group of 10–12 participants at risk for stroke, spans over 10 weeks and includes six 90-min group sessions with different foci augmented by an app. There are themes for each session and include stroke risk and engaging everyday activities (EEA), physical activity, diet, stress and activity balance. Each session consists of an information part, trying out an activity, and interaction and discussions between group members. In-depth information on the MMD intervention can be found elsewhere [34]. The MMD intervention has been feasibility- and pilot-tested [32, 36] and is now undergoing a Randomised Controlled Trial (RCT) (ClinicalTrials.gov: NCT05279508).

Despite progress in the area, there is a lack of guidance on how to undertake a co-creation process when developing, evaluating, and refining an intervention [29, 40]. This can contribute to identified gaps in relation to stakeholder engagement along certain criteria [9], however it can also be important to explore the potential utility of co-creation along a continuum of engagement in different parts of a process. Moreover, there is a dearth of literature on engaging stakeholders in the later stages of the research process, particularly in refining the intervention [29]*.* Hence, there is a need to understand in what manner refinements of an intervention can be identified and co-created with stakeholders in the later stages of the research process. The current study employs a co-creation method iteratively within an intervention study, in parallel with the RCT, through a cycle of refinements. This study aims to describe and explore the refinement of the MMD intervention and conditions for implementation in primary healthcare by utilising co-creation with stakeholders.

## Design & methods

**Fig. 1:**
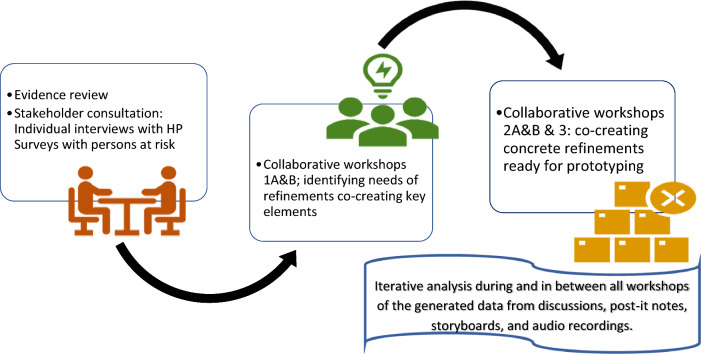
A flowchart of the co-creation process of this study guided by Hawkins et al. [15] three stages

To answer the aim this co-creation study engaging stakeholders in an iterative co-creation process was conducted. There is inconsistent use and varying definitions of terminology in collaborative studies, such as co-creation, co-design, and co-production [38, 51]. In this study, we followed Perez Jolles et al. [40] principles of “all things co” and utilised the concept of co-creation. Where meaningful stakeholder engagement sharing their unique knowledge, experiences and resources renders an end-result exceeding the sum of its parts [40]. The co-creation process in this study (Fig. 1) was guided by Hawkins et al. [15] three stages of co-creation, which include an evidence review and stakeholder consultation, co-creation, and prototyping.

### Stakeholder engagement

In previous stages of the MMD development, including an evidence review and a feasibility study, stakeholders were involved in identifying the need for a successful stroke prevention intervention in primary healthcare, establishing the foundation of the MMD intervention. Stakeholders included general practitioners, other experts in areas such as stroke, occupational therapy, and physical activity, decision-makers, and individuals at risk for stroke [35, 37]. For this study, stakeholders included HP delivering the intervention and individuals at risk for stroke who had received the MMD intervention. The engagement of stakeholders in an iterative process of co-creation was to understand their needs and behaviours, identify and explore relevant ideas, and co-create refinements [33]. According to the Spectrum of Public Participation [16], which ranges from Inform to Collaborate to Empower, this study is in the range of collaboration. Meaning the stakeholders were collaborators in refining the intervention while researchers made initial planning and final decisions [40]. The study has been approved by the Swedish Ethical Review Authority (Dnr:21-05902-02). In accordance with the Declaration of Helsinki. All participants were given both oral and written information about the study, its purpose, and design and given the possibility to ask questions. Written informed consent was obtained from all participants before inclusion.

### Stakeholders and settings

**Fig. 2:**
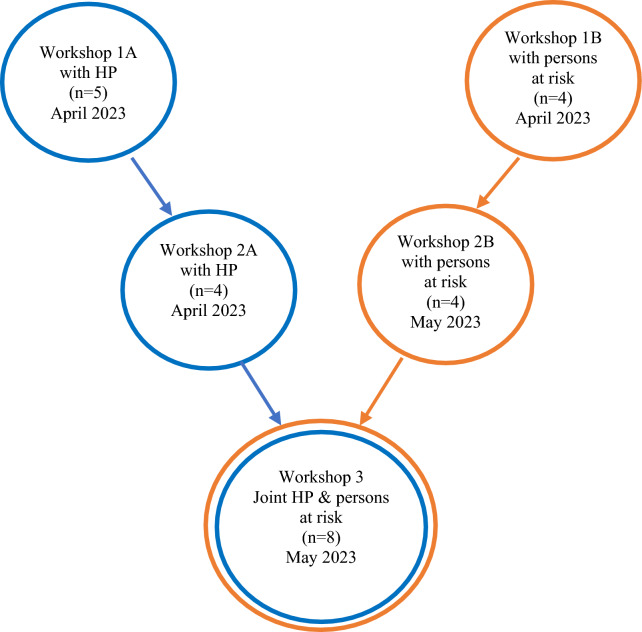
Flowchart of the workshop series of at total of five workshops with HP and persons at risk

Stakeholders were asked to participate in collaborative workshops focused on potential refinements needed to optimise implementation of the MMD intervention. In 2022–2023, ten HP’s delivered the MMD intervention to five cohorts as part of the MMD full-scale RCT. The ten HP’s, based at three primary healthcare clinics in the Stockholm region, including four occupational therapists (OT), three physiotherapists (FT), and three dieticians (D), were invited to participate in three workshops. Seven HP accepted the invitation. Persons at risk for stroke receiving the intervention in the first cohort of the MMD full-scale RCT in 2022 and who had completed the 12-month follow-up, a total of seven persons, were invited to collaborative workshops. The seven persons at risk for stroke were asked to participate in three workshops, with four accepting the invitation. Additionally, the two persons who had previously participated in the pilot RCT and development phases of the full-scale RCT were invited and accepted to participate. A total of 13 stakeholders participated in the workshops with a variation of stakeholders and number of stakeholders in each workshop. Not all HP or persons at risk had possibility to participate in every workshop; the number of participants in each workshop is outlined in Fig. 2. The number, length, dates and location of workshops were planned in accordance with the stakeholder groups’ preferences. The stakeholders and researchers were not familiar with each other, except from having met for information sessions and inclusion in the MMD intervention and study.

### Design of workshops

Workshops were designed based on a summary in the form of themes and vignettes based on literature and collected insights from prior phases of the MMD project, for example, stakeholder consultations (Fig. 1). A set of five collaborative workshops totally was conducted with the stakeholders. All workshops were iterative co-creation processes [15], entailing data generation and analysis. The workshops took place between April and May of 2023. The researchers designed the workshops and purposefully separated HP from the persons at risk for stroke in the initial four workshops (Fig. 2). This was done to establish a secure environment for shared experiences within each group and to minimise any potential challenges grounded in power dynamics before merging the two groups in the final workshop [7]. The duration of the workshops with persons at risk was 3 h, while the workshops with HP and the joint workshops were 2 h. Researchers and stakeholders viewed 3-h workshops as a reasonable length. However, due to time limitations for HP, the workshops they attended were shortened to 2 h. All workshops were onsite and face-to-face at the three different PHC clinics delivering the intervention, respectively. The participants were informed of and consented to the workshop's arrangement.

### Data generation—collaborative workshops

The process and goals of each workshop were stated by the researchers and agreed upon at the beginning of the workshop, as lack of clarity can jeopardise the success of co-creation processes [12]. Strategies such as clear purpose, frequent feedback, open communication, data sharing, and process transparency were utilised to ensure clarity of mutual expectations [1], 12. The first (CJ) and last (EA) authors planned and facilitated all five workshops. Both have in-depth knowledge of the MMD intervention and HP education. Both are occupational therapists, CJ with long and recent clinical experience in rehabilitation and EA with a long experience as an educator and as a researcher using co-creation methods and facilitating workshops.

**Fig. 3:**
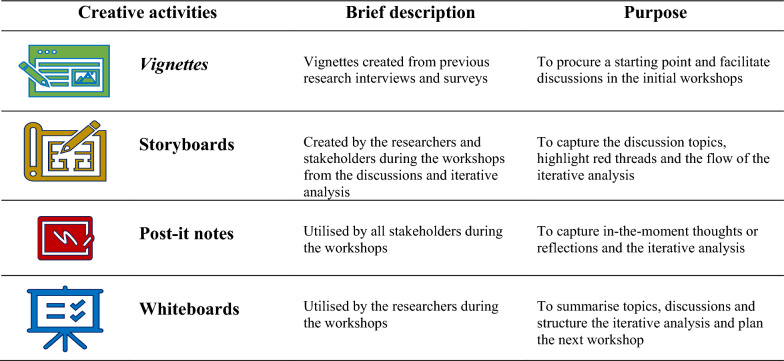
Creative activities utilised by stakeholders and researchers in the workshops for data generation and the iterative analysis

Topical themes and vignettes, developed in a prior stage of the MMD project by the first (CJ) and last (EA) author, were presented during the first workshop with HP and persons at risk, respectively (Table 1). During the first workshop, the stakeholders were given the opportunity to discuss and add to the themes. Both groups agreed with the presented themes. The themes and vignettes (see Appendix 1 and 2) were used to inform the co-creation process during the first workshop to empathise and define stakeholders' needs and problems within the MMD intervention [27]. Vignettes, outlined in Table 1, were snapshots describing a reflection in relation to the themes and utilised to facilitate discussion [28, 45] in the initial workshops. Through collaborative discussions around the defined problems, key components within the MMD intervention in need of refinements were identified and defined during the workshops. Subsequent workshops focused on ideating, generating, and creating concrete refinements addressing the identified key components through an iterative co-creation process. Different creative activities outlined in Fig. 3 were utilised during the workshops to engage the stakeholders and facilitate discussions [42] and the analysis. In the final workshop, stakeholders reached a consensus concerning refinements of the key components by reexploring key components and refinements. Each workshop was audio recorded and fieldnotes were taken.

**Table 1:** The starting point of the first workshops for HP and persons at risk, respectively

The HP workshop				
Themes	Team spirit in the HP groups	The importance of time	The diverse experience of HP education	The group; homogenous vs heterogenous
Vignette	Everyone did their part and had little insight to the other sessions and did not work together as a team	Allocation of time during the sessions—> The use of time and planning for the sessions and the education	Feelings of uncertainty in the set-up, to present and give feedback to each other	There was a large diversity in the group; all had a risk for stroke, but there was a variation in risk levels and prerequisites
Vignette	A feeling of team spirit with joint discussions on sessions and concepts	Consent of time (use) from manager	Appreciation of the disposition and pedagogy—a good learning opportunity before execution	The participants were interested, had a lot of questions, and shared motivation

The persons at risk workshop				
Themes	The group; homogeneity vs heterogeneity	Defining & relating EEA to everyday life activities	Goal fulfillment augmented through the sessions	The app, a facilitator or barrier in the change process
Vignette	Difficult when the physical prerequisites differ greatly between participants	Perceived as hard to grasp and to include in everyday life	The sessions did not fully support the process towards set goals	Frustrating to use, took a lot of time, several malfunctions and do overs
Vignette	Rewarding and safe to meet people in the same situation	A novel and interesting way to think of ones everyday activities	Formulating goals was helpful in the process	Has potential to be a useful tool to support change

The themes and vignettes were created in the first stage from a stakeholder consultation

### Data analysis—collaborative workshops

The workshops, involving both researchers and stakeholders, were used to inform an iterative analysis. The stakeholders were active in the iterative analysis, facilitating a shared understanding of the objectives and identifying and defining key components and refinements. Both stakeholders and researchers utilised creative activities, such as post-it notes and storyboards, aiding the iterative analysis and creation of refinements during the workshops (Fig. 3). The stakeholder groups outlined common areas and topics in the data by viewing and analysing all data using creative activities (whiteboards, post-it notes, storyboards), and through discussions. The process was revisited in each workshop, informing the discussions and further analysis, grouping the data, and identifying key components. The HP and the persons at risk identified similar key components independently. The first (CJ) and last (EA) authors concluded each workshop with a brief summary of the discussion and take-home messages verified by the stakeholders, which served as a foundation for the following workshop [24]. After each workshop, the first author (CJ) wrote comprehensive fieldnotes and compiled data from the creative activities based on group discussions, analysis and workshop reflections. The first author (CJ) also listened to each session's audio recordings to identify gaps between topics discussed and recorded on the whiteboard and post-it notes. In preparation for the joint workshop, the data from previous workshops were carefully processed and compiled by the first (CJ) and last (EA) authors, presented to the stakeholders verbally, and outlined on whiteboards. When compiling the key elements, the first (CJ) and last (EA) authors identified the pedagogy of MMD aligning the key elements. The workshop series was concluded when no new insights or reflections were brought to light. Final refinements were revisited to ensure alignment with the study's objectives.

## Results

Co-creation workshops with stakeholders generated valuable insights into the stakeholders' experiences of the MMD intervention. Four key components were identified through the co-creation process. *The pedagogy of MMD,* which was overarching, and three more key components: *the HP education*, *the MMD intervention sessions*, and *the digital tool*. In this study, the pedagogy refers partly to the learning model and peer coaching approach used in the HP education (Jakobsson, in revision) and partly to the coaching approach employed by the HP delivering the MMD intervention, including the app. Refinements addressing these key components were co-created. The result will be presented chronologically (Fig. 2), with the last joint workshop summarising the co-created refinements.

### Workshops with HP

In the first workshop with HP, shared experiences led to identifying pedagogy of MMD as a central component on which to focus. The key component was identified from themes *of team spirit, the importance of time, and the HP education*. Discussions focused on what HP felt was lacking, what they would have needed, what worked well, and what could be further built on. It was perceived differently in the HP group, where some enjoyed the overall pedagogical approach while others struggled. In the second workshop, alternative approaches to the pedagogy of MMD, both HP education and intervention delivery, were collaboratively discussed. The HP education content and material was appreciated and perceived as highly relevant among HP. However, they felt they would have benefitted from more time to reflect and discuss, deepen their understanding of the material and concepts, and collaborate as a team. Ideation resulted in suggestions involving a more structured, collaborative and focused two-day education, actively engaging the HP’s in all topics and sessions of MMD. Moreover, to boost motivation and add more credibility to the education and intervention, it was suggested that the HP education should include some sort of calibration, resulting in a diploma and credits. Additionally, the group considered refining the HP education as a strategy to address the theme of team spirit. Team spirit could be enhanced by including more time to work together as a team and participate in the entire MMD intervention instead of only in siloed modules pertaining to one’s own professional competency area. An explicit refinement was having two HP’s deliver each session in order to facilitate a shared understanding of participants' discussions, goals, and change processes. Additionally, the suggested refinement also addressed the importance of time, as shared knowledge was perceived to save time in organizing, preparing, and carrying out the intervention. (Table 2).

**Table 2:** An overview of the process of co-creating the refinements

Outputs on refinements from the workshops			
From workshop 1A&B The What; Emphasise & Define		From Workshops 2A&B & 3 The How; Define & Ideate	
		Co-created suggestions for Refinements	
Key components	The now	Workshops 2 A& B	Workshop 3
1) Pedagogy; The HP education	Education package on the digital platform Canvas with reading material. 2 digital and 1 face-to-face meeting, Tot 8 h	A face-to-face MMD course, 1–2 course days and 3 academic days Team collaboration Requirement specification	Course material for two course days. Keep the same material as now but, more time allocated for concepts and delivery Interprofessional focus pervading Calibration & diploma. 1credit
2a) Pedagogy; The MMD sessions for persons at risk for stroke	6 on-site group sessions with lectures from HP, group discussions and reflections, and try-out activities	Prerecorded lecture with concluding quiz Scientific article Popular science summary Starting segment—how the week was, goals and EEA Several small group discussions Closing segment—summary of the session and it discussions	Videos with the lectures + quizzes Scientific articles and popular science summary for each occasion and theme A group segment reflecting on EEA and goal attainment from the past week Variation of small group constellations of 3-5p during the sessions discussion topics A group segment reflecting on EEA and goal attainment from the topic of the session
2b) Pedagogy; The MMD sessions for HP	Feeling secure in delivering the material. Peer coaching in the delivery of the intervention Lecture on pre-set topic for the session Moderate discussions and a try-out activity HP 1/session	A MMD course for HP containing the core concepts and pedagogy of MMD Other pedagogical approaches with pre.rec lecture. Summarise lectures with a quizz Moderate the starting and closing segment Moderate small group discussions Hold try-out activities All HP be more involved & engaged. Possibility to be 2 HP	Focus on knowledge of stroke risks, core concepts: EEA, activity balance, habits, and motivation, facilitating reflection About 30 min prerecorded + 60 min group. Punctuate key points for summary and quiz answers, discussion points and topics Cues for the starting and closing segment focusing on EEA and goal attainment Themes to facilitate small group discussions Boxes with activities to select for each session Different constellations of 2 HP in each session
3) Pedagogy: The digital tool	An App to register daily on diet, physical activity, EEA and goal achievment	Website—digital connection with the group during and after the intervention	Website with personal login Registering and prompts Library Prerecorded films Scientific articles and popular science summary

First, understanding the current MMD and identifying key components in need of refinements. Second, co-creation and refinements of MMD

### Workshops with persons at risk for stroke

During the first workshop with the persons at risk for stroke, the importance of the key component of *MMD intervention sessions* was identified as well as an overlap with the importance of pedagogical approaches used in MMD. The key component was identified from the themes of *the diversity of the group, defining and relating engaging everyday activities to everyday activities,* and *goal fulfilment augmented through the sessions* collectively (Table 1). The persons at risk for stroke felt that the content of MMD supported their change process. However, they expressed how there was room for improvement regarding the structure and delivery. During the second workshop, suggestions for refinements were ideated. The focal points of the discourse revolved around knowledge and structure. Emphasis was on augmenting the delivery of the MMD intervention and, as a consequence, affecting how the MMD intervention was received. The interventions intended alignment of engaging everyday activities and work towards pre-set goals were not always made clear and, therefore, described as sometimes difficult to grasp and relate to one’s own everyday life and engaging activities*.* The stakeholders collaborated on ideas enhancing clarity and offering practical solutions to refine the pedagogy of the MMD intervention. A suggested refinement was to incorporate a distinct starting and concluding segment in each session of the MMD intervention. They expressed that HP should facilitate the segments and focus on motivating and prompting participants to stay on track with their goals and reflect upon engaging everyday activities in their everyday lives. Group discussions and sharing experiences were believed to enhance the understanding of engaging everyday activities and to encourage the work towards goal fulfilment. To address this, a concrete refinement was having several small group discussions on pre-set discussion topics during the session. (Table 2).

The theme *the app* received mixed reviews among the group, and *the digital tool* was defined as a key component. Some found the app a facilitator, providing support and guidance, while others found it a barrier, adding extra work to their already busy schedules. As the group discussed the digital tool, they ideated numerous ideas on how it could better serve as a facilitator and support system for making lifestyle changes. Among the suggested refinements was having a digital platform rather than just an app, accessible through various devices with a login. While the group didn’t come to a unanimous decision on the specifics, there were a few points that everyone agreed upon. These included registering and receiving feedback on personal goals, tracking goal-relevant information and engaging everyday activities, and accessing MMD-related articles and resources (Table 2).

### Joint workshop with HP and persons at risk for stroke

During the fifth and final joint workshop, the suggestions for refinements made in the prior workshops were revisited. The stakeholders collaborated on more detailed suggestions for refining the key components, as outlined in Table 2. These refinements aligned with the overall aim of the co-creation process: to identify and co-create refinements in MMD to refine the intervention for its stakeholders and future implementation.

From the perspective of HP, detailed suggestions for refinement of the key component *the pedagogy of the HP education* entailed a structured, situation-bound face-to-face education held by MMD experts. This approach would enable HP’s to acquire and enhance their knowledge and skills on core concepts, such as engaging in everyday activities, and the pedagogy of MMD in a more methodical manner than previously. Additionally, it would cover how to deliver the MMD intervention collaboratively as an interprofessional team and actively engage in all aspects of MMD. Reflecting and discussing with other HP attending the education and the experts were believed to facilitate a greater understanding of MMD. The collaborated suggestion of two full days for the HP education was thought possible regarding the clinic prerequisites. The benefit of calibration and credit-rendering certificate of completion for the HP education was emphasised by the HP’s. They also recognized that the proposed refinement, involving the collaboration of two HP’s in each session, further promoted the intended interprofessional approach for MMD."

From the perspective of the persons at risk for stroke, discussions about the key component *the pedagogy of the MMD sessions* rendered suggestions for refining the structure and delivery of the intervention. They highly valued the opportunity for reflection and discussions within the group and with the HP, proposing that more time could be allocated for these activities. One suggestion was to provide pre-recorded lectures on the web platform prior to the group session to rearrange the information portion of the course. This would allow for more time devoted to group reflections, discussions, and the activity. Another concrete refinement proposed was to incorporate a starting and closing segment in each session dedicated to engaging everyday activities and goal attainment. This was intended to enhance understanding, reflection, and integration of incorporating everyday activities into everyday life, and to promote and reflect on progress towards individual goals. These segments would involve HP guiding the discussion, referencing the previous week's session and participants' own efforts. The persons at risk co-created reflection prompts such as working towards one's goals, handling setbacks, and sharing engaging everyday activities.

The stakeholders jointly co-created concrete discussion cues for the small group discussions. The cues encompassed what makes this activity an engaging everyday activity for you, the facilitator and barriers in everyday life for goal attainment, strategies for goal diversity, and experiences of physical activity or snack preparation. Additionally, a list of activities to choose from in the experienced-based activity was co-created, depending on the group's prerequisites and wishes. Examples of activities included one-on-one talk on engaging everyday activities, qi-gong, nature walks, gym exercises, walk and talk on goals and strategies, circle phys and snack preparations. The persons at risk for stroke recognised the value of the HP's educational efforts to establish a shared language and approach among the HP. How this could have a positive impact on the implementation and delivery of the intervention. Moreover, it was recognised as a possible facilitator for the suggested starting and closing segments.

For refinements concerning the key component of *the digital tool,* the concrete suggestions made in the prior workshop with persons at risk for stroke of a digital platform and its potential content were revisited. There was a consensus on the benefits of a digital platform over an app. The diversity in the group brought forth varied needs and content also in this workshop, rendering no new suggestions or consensus.

## Discussion

The goal of stakeholder engagement in this study was to identify and refine key components in the MMD stroke prevention intervention. Through the co-creation process, four key components to refine were identified: 1) *the HP education*, *the pedagogy used in the MMD program,* both for *2a) persons at risk for stroke* and 2b) *health professionals, and 3) the digital tool.* The co-creation process contributed to refining the intervention targeting health behaviour change, which is in line with other co-creation studies [4, 25, 27, 30, 39, 42]. Moreover, it will inform the next phase in optimising the MMD intervention for implementation in primary healthcare. Furthermore, this study fills a gap in demonstrating advantages of engaging stakeholders in an iterative co-creation process even in later stages of the research process.

### Co-creating the refinement of components

Both HP and the persons at risk emphasized the importance of a confident, reflective, and interactive delivery grounded in the interventions’ main concepts. The co-creation process informed a refined HP education with foci enhancing concept knowledge, team collaboration, a feeling of material ownership, and a calibration for motivation and commitment. Studies have identified that understanding the stakeholders' perspectives is imperative to enhance the meaning and relevance of new knowledge [38] and acceptability of co-created content [25]. The persons at risk discussed how the delivery of the intervention affected their understanding and, to some extent, perceived relevance of the concepts within the intervention. This aligns with the idea that ownership can contribute to a commitment and increased implementation effectiveness of the intervention in practice [23] and motivation among stakeholders [13, 40]. Conversely, it has been reported that HP's low confidence in their knowledge and competence can negatively impact the delivery and, consequently, the acceptability for those receiving the intervention [3]. The HP’s allocated time to, perception of, and commitment to the HP education varied. Additionally, the HP’s perceived varying levels of support from their employers and clinical structures regarding preparation time, team discussions and time to devote to the HP education. These factors affected their perception of ownership and commitment to delivering the MMD intervention and prompted refinements of the HP education, such as two set course days rendering continuing education credit and a diploma.

### Refining interprofessional team work in the MMD intervention

The MMD intervention, with its alignment of engaging everyday activities and goal fulfillment, requires interprofessional team collaboration to foster participant confidence and motivation. Based on the results, the research team contemplated whether they had too naively presumed that the PHC teams worked interprofessionally when, in practice, it often appeared to be multi-professionally as parallel professional work [18, 48]. The workshops exposed how HP's ambitions or desires to work together collaboratively did not match the ambitions of interprofessional work in the clinic or existing primary healthcare structures. Studies have shown that there often is a lack of clinical routines, structure and tools to carry out interprofessional stroke prevention [21, 26, 37]. Hence, an intervention such as MMD could be one tool facilitating interprofessional prevention work in PHC. As lifestyle interventions target the complexity of risk factors, an interprofessional approach is seen as necessary for the optimal delivery and acceptability of the intervention [3, 5, 20, 48] and facilitator for behaviour change [31].

### Method discussion

A strength of this study is its novel approach, utilising an iterative analysis during the co-creation workshops in which the stakeholders actively engaged in the analysis. The iterative analysis rendered committed stakeholders and stakeholder-relevant results. The authors find, to date, no previous intervention studies reporting on utilising co-creation and an iterative analysis method with the stakeholders under and in between workshop. However, one study describes the successful use of an iterative approach and co-productive workshops, but with the analysis performed by the researchers after the workshops [14]. Moreover, a systematic review found that the flexibility of utilising an iterative cyclic process in co-creation enhances trust and a shared understanding of the solutions [13]. The results of this study may inform the design of co-creation in future intervention studies. In this study, we were guided by the three stages of Hawkins et al. [15]. The first stage, stakeholder consultation, conducted prior to this study, procured a valuable and informed foundation for stage two, the co-creation workshops. The iterative collaborative process described by Hawkins et al. [15] in stage two facilitated fruitful discussions in the workshops and the identification of key elements and co-creation of refinments. An important aspect of the co-creation process in this study is its position on the Spectrum of Public Participation (IAP2, 2015; [40]), namely collaboration. In this study, stakeholders were engaged in intervention refinements, and the goal was not to re-design the MMD intervention, but rather to increase knowledge on how to improve the interventions identified key components to a later stage of implementation. As researchers, we ultimately decided whether to proceed with a co-created refinement based on its alignment with the purpose of MMD. This can be critiqued as lacking a more engaged level of patient and public involvement and is something that the research team is considering as future studies are planned. Also, the number of stakeholders participating in the workshops could be a limitation to the study. However, the broad mix of allied health professionals with a varying degrees of professional years, mix of men and women and the different background of the stakeholders, all contributed to fruitful discussions and the iterative co-creation process. The results show that there is value in engaging stakeholders in the intervention refinements. Moreover, co-creation processes are time intensive, and in this study, we could not move through all phases, including the last stage in Hawkins et al. [15] three stages, testing prototypes. This is a limitation that needs to be balanced with time commitments among stakeholders and potential benefits for outcomes in the project. In this case, the team considered that the potential benefits of balancing all stakeholder commitments were justified in moving along the development of the refined MMD intervention.

### Roles and power dynamics

An imperative consideration and potential weakness in conducting this study was the power dynamic between stakeholders and researchers [7]. The workshops consisted of the HP, the persons at risk for stroke, and two researchers. The researchers had been part of developing and evaluating the MMD intervention and the HP education. There was a risk that our experiences would lead to over- or underinterpretation of the data and discussions. An active strategy was to remain open about our respective backgrounds and experiences with stakeholders and to reflect on whether our experiences influenced or steered the conversations in any way [33]. Having previously held various roles within MMD, all stakeholders now brought their unique perspectives to the co-creation process. However, when engaging with stakeholders from diverse experiences, there is a risk of role ambiguity, disagreements, and varying levels of trust [1]. The decision of whether to mix or separate stakeholder groups during workshops was complex, as it involved weighing the benefits of gaining diverse perspectives against the potential risk of breaching privacy or causing harm. However, separating groups may lead to less productive conversations and, ultimately, a less successful outcome with potential ethical implications. Studies report having a purposive sample and smaller groups builds the group dynamic, facilitating the co-creation process [7] and facilitate following larger group discussions [25].

## Conclusion

Co-creation with stakeholders led to concrete refinements of key components in the MMD intervention and the HP education. The refinements are representative of the stakeholders, enhancing the relevance and acceptability of the intervention and optimising it for future implementation in PHC. Through the co-creation process, the significance of the pedagogy in a complex intervention was revealed. The process underscored the need to augment material ownership and to improve interprofessional collaboration in PHC, ultimately enhancing the intervention experience and facilitating the change process for individuals at risk of stroke.

## Data Availability

The datasets generated and/or analysed during the current study are not publicly available due protection of identifiable information on person-level, also regulated by our national ethics authorities, but are available from the corresponding author on reasonable request.

## Appendix 1

Vignette workshop 1A, HP.

## Appendix 2

Vignette workshop 1B, (persons at risk).

## Abbreviations

MMD: Make My Day
HP: Healthcare professionals
EEA: Engaging everyday activities
OT: Occupational Therapist
FT: Physiotherapist
D: Dietician
PHC: Primary Healthcare
MRC: Medical Research Counsil
RCT: Randomised Controlled Trial
